# Dual-probe molecular MRI for the *in vivo* characterization of atherosclerosis in a mouse model: *Simultaneous assessment of plaque inflammation and extracellular-matrix remodeling*

**DOI:** 10.1038/s41598-019-50100-8

**Published:** 2019-09-25

**Authors:** Carolin Reimann, Julia Brangsch, Jan O. Kaufmann, Lisa C. Adams, David C. Onthank, Christa Thöne-Reineke, Simon P. Robinson, Bernd Hamm, Rene M. Botnar, Marcus R. Makowski

**Affiliations:** 1 Charité – Universitätsmedizin Berlin, corporate member of Freie Universität Berlin, Humboldt-Universität zu Berlin, and Berlin Institute of Health, Charitéplatz 1, 10117 Berlin, Germany; 2 Department of Veterinary Medicine, Institute of Animal Welfare, Animal Behavior and Laboratory Animal Science, Freie Universität Berlin, Königsweg 67, Building 21, 14163 Berlin, Germany; 3 Federal Institute for Materials Research and Testing (BAM), Division 1.5 Protein Analysis, Richard-Willstätter-Str. 11, 12489 Berlin, Germany; 4 Humboldt-Universität Berlin, Department of Chemistry, Brook-Taylor-Str. 2, 12489 Berlin, Germany; 5 Lantheus Medical Imaging, North Billerica, Massachusetts, 331 Treble Cove Road, USA; 6 King’s College London, School of Biomedical Engineering and Imaging Sciences, St Thomas’ Hospital Westminster Bridge Road, London, SE1 7EH United Kingdom; 7 Wellcome Trust/EPSRC Centre for Medical Engineering, King’s College London, London, United Kingdom; 8 BHF Centre of Excellence, King’s College London, London, United Kingdom; 9 Escuela de Ingeniería, Pontificia Universidad Católica de Chile, Santiago, Chile

**Keywords:** Molecular medicine, Experimental models of disease

## Abstract

Molecular MRI is a promising *in-vivo* modality to detect and quantify morphological and molecular vessel-wall changes in atherosclerosis. The combination of different molecular biomarkers may improve the risk stratification of patients. This study aimed to investigate the feasibility of simultaneous visualization and quantification of plaque-burden and inflammatory activity by dual-probe molecular MRI in a mouse-model of progressive atherosclerosis and in response-to-therapy. Homozygous apolipoprotein E knockout mice (ApoE^−/−^) were fed a high-fat-diet (HFD) for up to four-months prior to MRI of the brachiocephalic-artery. To assess response-to-therapy, a statin was administered for the same duration. MR imaging was performed before and after administration of an elastin-specific gadolinium-based and a macrophage-specific iron-oxide-based probe. Following *in-vivo* MRI, samples were analyzed using histology, immunohistochemistry, inductively-coupled-mass-spectrometry and laser-inductively-coupled-mass-spectrometry. In atherosclerotic-plaques, intraplaque expression of elastic-fibers and inflammatory activity were not directly linked. While the elastin-specific probe demonstrated the highest accumulation in advanced atherosclerotic-plaques after four-months of HFD, the iron-oxide-based probe showed highest accumulation in early atherosclerotic-plaques after two-months of HFD. *In-vivo* measurements for the elastin and iron-oxide-probe were in good agreement with *ex-vivo* histopathology (Elastica-van-Giesson stain: y = 298.2 + 5.8, R^2^ = 0.83, p < 0.05; Perls‘ Prussian-blue-stain: y = 834.1 + 0.67, R^2^ = 0.88, p < 0.05). Contrast-to-noise-ratio (CNR) measurements of the elastin probe were in good agreement with ICP-MS (y = 0.11x-11.3, R² = 0.73, p < 0.05). Late stage atherosclerotic-plaques displayed the strongest increase in both CNR and gadolinium concentration (p < 0.05). The gadolinium probe did not affect the visualization of the iron-oxide-probe and vice versa. This study demonstrates the feasibility of simultaneous assessment of plaque-burden and inflammatory activity by dual-probe molecular MRI of progressive atherosclerosis. The *in-vivo* detection and quantification of different MR biomarkers in a single scan could be useful to improve characterization of atherosclerotic-lesions.

## Introduction

Atherosclerosis is the leading cause of morbidity and mortality in the Western world and developing countries^1^. The development of atherosclerosis is a complex process with a dynamic influx of proinflammatory cells and a progressive increase of plaque-burden, which is associated with de-novo synthesis of extracellular-matrix proteins^2^.

Atherosclerosis progresses relatively slowly and, in most cases, remains asymptomatic until the end-stage of the disease. Sudden surface erosion or plaque rupture can lead to vascular occlusion and ischemia, resulting in myocardial infarction or cerebral stroke^3^.

In a clinical setting, conventional angiographic techniques are used to assess the degree of luminal narrowing in the coronary and carotid-arteries^4^. Even though angiographic techniques are the diagnostic reference standard, there is growing evidence, that the interventional treatment of the atherosclerotic-plaque, associated with the highest degree of stenosis, does not improve the prognosis in all patients^5^. Furthermore, previous research demonstrated that the highest number of ruptured atherosclerotic-plaques were found in arteries with <60% of stenosis^6^.

Substantial progress has been made with invasive and non-invasive imaging methods in the quest for the “vulnerable plaque” in recent years. It is well established, that features of vulnerable plaques go beyond the severity of vascular stenosis^7^. There is a combination of plaque features, which are strongly associated with plaque vulnerability, including the influx of proinflammatory cells, positive vascular-remodeling, angiogenesis, a large necrotic core, a thin fibrotic cap, micro-calcifications and plaque hemorrhage.

In this context several invasive and noninvasive imaging techniques have been investigated, but none has been widely adopted in in clinical practice.

Inflammatory activity and plaque-burden, which can both be visualized by molecular MRI techniques, were established as key features of vulnerable atherosclerotic-plaques. Therefore, the combined assessment of two different plaque characteristics in one scan has the potential to improve the stratification of patients at risk for myocardial infarction or cerebral stroke^8^. So far, a combined assessment of two features (inflammatory activity, plaque-burden) in a single scan has not been established in the context of atherosclerosis. This study aimed to investigate the feasibility of a simultaneous characterization of the plaque-burden and inflammatory activity by dual-probe molecular MRI in a mouse-model of progressive atherosclerosis and in response-to-therapy. For the visualization of the extracellular-matrix we used a gadolinium-based elastin-specific agent, for the visualization of proinflammatory cells we used a clinically approved iron-oxide agent Feraheme (Ferumoxytol, AMAG-Pharmaceuticals, Waltham, MA, USA). There are a couple of applications with simultaneous use of Gd-based and iron-oxide -based imaging probes. They demonstrate that high-resolution magnetic resonance imaging (MRI) allows combining cellular-scale resolution with the ability to detect two cell types simultaneously at any tissue depth^9,10^.

## Materials and Methods

### Animals

This study was performed according the local guidelines and provisions for the implementation of the Animal Welfare Act and regulations of the Federation of Laboratory Animal Science Associations (FELASA). The regulatory authority the Regional Office for Health and Social Affairs Berlin (LAGeSo) approved this animal study. We used eight weeks old apolipoprotein-E-deficient (ApoE^−/−^) male mice. ApoE^−/−^ mice were fed with a high-fat-diet (HFD) containing 21% fat from lard, supplemented with 0.15% (wt/wt) cholesterol (Special-Diets-Services, Witham, UK) at the age of two-months. A control group with nine 26 week old male C57BL/6J mice was used. Statin-treatment was performed in a subgroup of ApoE^−/−^ mice (n = 9) and started in parallel with the feeding of the HFD feeding. Pravastatin (Kemprotec-Limited, Middlesbrough, UK) was administered at a dose of 40 mg/kg body-weight/day via drinking water. Please refer to Supplementary Fig. 1 for the experimental setup and for further details to the Supplementary Material.

### Gadolinium-based elastin-specific agent

In this study we used a low molecular weight gadolinium-based (856 g/mol molecular-mass) elastin-specific MR agent. A rapid clearance from the blood-pool via the kidneys and highest binding can be measured 30 to 45 minutes following the administration^11,12^. For the unbound and bound agent, a longitudinal-relaxivity of 4.68 ± 0.13 mM^−1^s^−1^ and 8.65 ± 0.42 mM^−1^s^−1^ was reported, which is higher than for clinical used agents, such as gadobutrol^11,12^. This probe was validated and used in different previous studies to visualize the extracellular-matrix (ECM) protein, elastin^11,12^. The *in-vivo* visualization of the ECM protein elastin in atherosclerotic-plaque represents a surrogate marker for overall plaque-burden^11,12^. In this study, the elastin-specific probe was administered *via* the tail vein at a clinically used dose of 0.2 mmol/kg on days one of MRI examination and after 24 h on day two of MRI examination.

### Iron-oxide-based macrophage-specific agent

The iron-oxide-particle used in this study is coated with semisynthetic carbohydrate (ferumoxytol). Because of its effectiveness in shortening T1 and T2 relaxation times this agent was developed as a MRI agent initially^13^. Ferumoxytol (Feraheme®, AMAG-Pharmaceuticals, Waltham, MA, USA) was approved by the Food-and-Drug-Administration (FDA) for iron-deficiency-anemia treatment in adults with chronic-kidney- disease (CKD) in 2009. In recent years the use of off-label iron-oxide nanoparticle as a MRI probe by clinicians and researchers has grown^13^. A linear dependence of R1, R2, and R2* on ferumoxytol concentration was found in saline and plasma with lower R1 values at 3.0 T and similar R2 and R2* values at 3.0 T (r1_saline_ = 10.0 ± 0.3smM; r1_plasma_ = 9.5 ± 0.2smM; r2_saline_ = 62.3 ± 3.7smM; r2_plasma_ = 65.2 ± 1.8smM; r2*_saline_ = 57.0 ± 4.7smM; r2*_plasma_ = 55.7 ± 4.4smM)^14^. In this study, ferumoxytol was administered at the clinical dose (4 mg Fe/kg) *via* the tail vein injection and imaging was performed after 24-hours.

### *In-vivo* MR Experiments

Imaging sessions were performed at a clinical 3T Siemens system (Biograph, Siemens Healthcare, Erlangen, Germany) with a single loop coil (diameter 4 cm) with mice in prone position. For details regarding the T1-weighted and T2*-weighted sequences please refer to the Supplementary Material.

### Assessment of magnetic resonance imaging signal

#### Evaluation of the gadolinium-based elastin-specific probe on T1-weighted sequences

The quantification of the MRI signal was conducted using OsiriX (OsiriX Foundation, Geneva, Version5.6). To assess the lumen and arterial-wall including the atherosclerotic-plaque, time-of-flight (TOF) images were automatically co-registered and overlaid with high–resolution MRI images. Morphometric measurements were implemented on high–resolution MRI images. Regions of interest (ROIs) were co–localized with the atherosclerotic-plaque (highest signal within the arterial wall) and defined as areas of enhancement on high–resolution MRI images. We calculated the contrast-to-noise-ratio with following equation: CNR = (Combined vessel-wall and atherosclerotic-tissue-signal − Blood-signal)/Noise. The noise was defined as the standard deviation in pixel intensity from a ROI placed in the background air anterior to brachiocephalic-artery.

#### Quantification of iron-oxide on T2*-weighted sequences

MR images of the vessel-lumen areas were compared between pre-contrast T2*-weighted MR images prior and iron-oxide injection 24 h post injection. All data originated from T2*-weighted images are demonstrated the signal-loss area (%) in relating to the pre-contrast MRI scan. To compare areas of signal-void within the ROI identical window and level settings were used. The areas were established semi-automatically using identic 2D-segmentation parameters.

### Histological analysis and immunofluorescence of the arterial vessel system and plaque morphometry

Please refer to the Supplementary Material.

### Inductively coupled mass spectrometry for quantification of gadolinium and iron and Laser-inductively-coupled-mass-spectrometry (LA ICP-MS) for spatial localization of gadolinium

Please refer to the Supplementary Material.

### Competition experiments

Please refer to the Supplementary Material.

### Statistical analysis

Please refer to the Supplementary Material.

## Results

### Multi-target plaque characterization in a single MR imaging session

#### Simultaneous imaging of the elastin-specific gadolinium-based probe and the macrophage-specific iron-oxide-based probe

**Figure 1:**
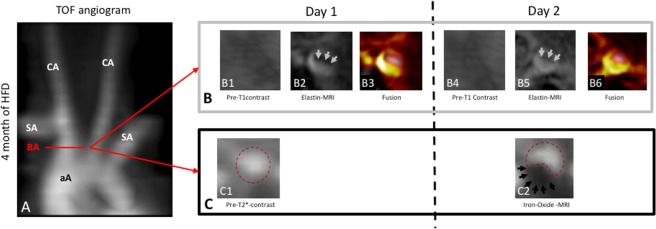
Multitarget characterization of the plaque-burden and inflammatory activity by molecular MRI. (**A**) Time-of-flight-angiography showing the aortic-arch, brachiocephalic-artery, subclavian-artery and carotid-arteries of an ApoE^−/−^ mice after four-months of high-fat-diet. (**B)** T1-weighted-imaging pre-contrast (B1, B4) and following the administration of the gadolinium-based elastin-specific MR-probe (B2, B5) at the clinical dose of 0.2 mmol/kg. Automatic image-fusion (B3, B6) demonstrates the localization of the signal from the MR-probe in the vascular-wall in relation to the blood signal (B4). (**C**) T2*-weighted-imaging prior to and following the administration of the iron-oxide-probe (ferumoxytol) at the clinical dose of 4 mg Fe/kg. On pre-contrast images, the vascular lumen can be clearly delineated for circular shape (C1). 24-hours following the administration of the iron-oxide-particle, a clear signal-void reduces the signal from the lumen can be visualized (C2), reflecting the accumulation of the iron-oxide-particles in atherosclerotic-plaques.

This study demonstrates that MR plaque-imaging with two probes can be performed in a single imaging session (Fig. 1). Using a combination of gadolinium-based elastin-specific imaging and iron-oxide-based macrophage-specific imaging different phases of atherosclerotic-plaque development and response-to-therapy could be evaluated. More specifically, de novo synthesis of elastic-fibers and inflammatory activity were not directly linked in atherosclerotic-plaques. In the early-stage of plaque development after two-months of high-fat-diet, only a small increase in de-novo elastin synthesis was observed (14% of the maximum after four-months of high-fat-diet) in the atherosclerotic-plaque. In contrast, a marked increase in iron-oxide-particle accumulation was measured (45% of maximum after four-months of high-fat-diet), indicating a strong inflammatory response in the early-stage of the disease. In advanced atherosclerotic-plaques,the influx of macrophages was most pronounced after four-months of high-fat-diet and in the most advanced plaques. In agreement with this abundant deposition of elastic-fibers in the plaque matrix was measured. This indicates, that in advanced plaques strong ECM-remodeling is accompanied by a large influx of pro-inflammatory macrophages.

In response to statin-treatment a different relative response was measured regarding the expression of elastic-fibers and the influx of macrophages. The amount of elastin detectable with ESMA was decreased by 43% compared to the four-months high-fat-diet group, while the influx of macrophages showed an even more pronounced decrease of 73% compared to the four-months high-fat-diet group.

### T1-weighted MR imaging for the assessment of the gadolinium-based elastin-specific MR-probe

**Figure 2:**
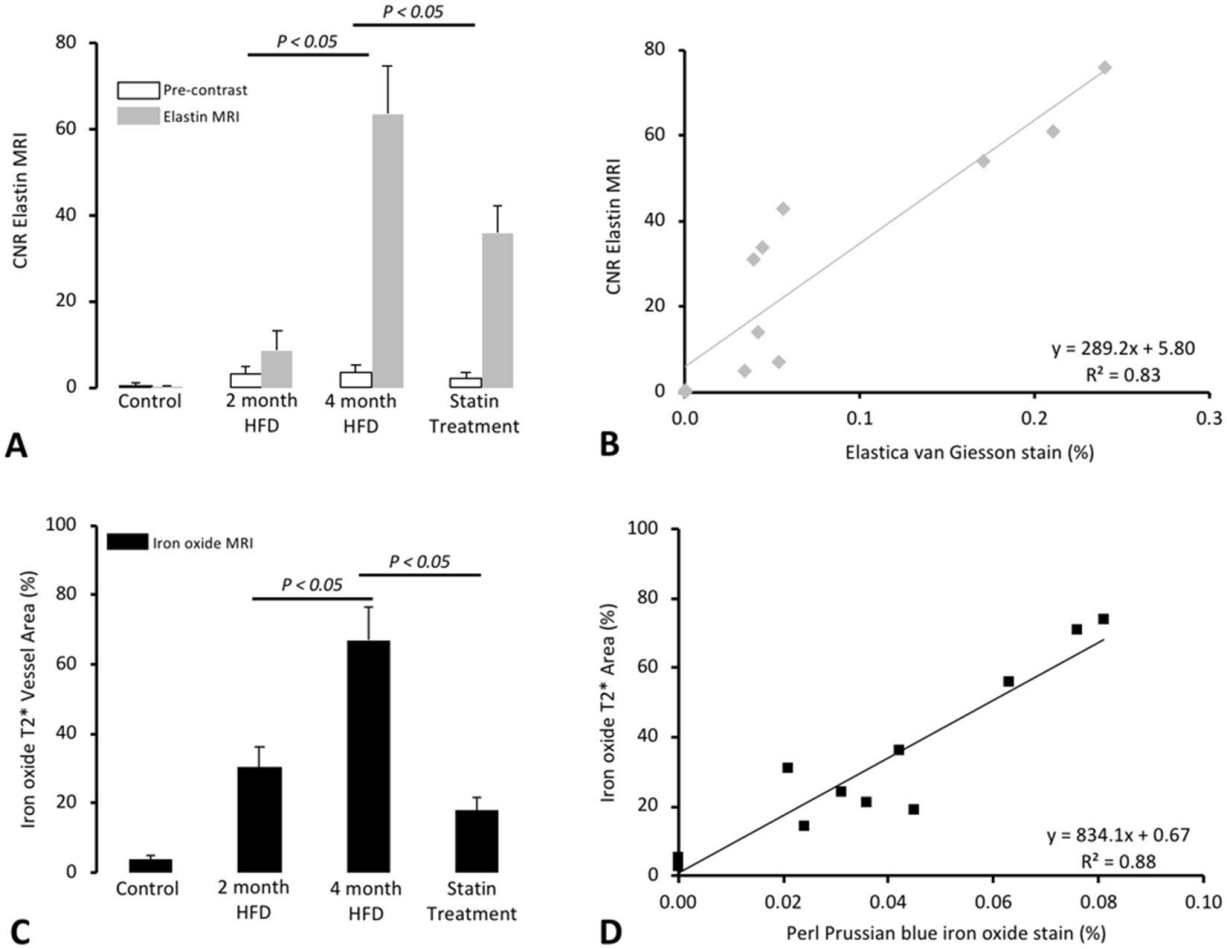
*In vivo* MRI signal measurements and *ex vivo* quantification of gadolinium-based and iron-oxide-based molecular probes. (**A**) Average atherosclerotic-plaque CNR pre–contrast and following the administration of the gadolinium-based elastin-specific MR-probe. After the injection of the elastin-specific probe, a small increase (p ≤ 0.05) in atherosclerotic-plaque CNR could be observed, while a strong increase in plaque CNR could be observed after four-months of HFD. (**B**) In the scatter-plot, the *in vivo* measured CNR showed a strong correlation (p ≤ 0.05) with the *ex vivo* Elastica-van-Giesson-staining. (**C**) After two-months of high-fat-diet a strong accumulation (p ≤ 0.05) of iron-oxide-particles could be observed. After four-months of high-fat-diet, the most pronounced peak (p ≤ 0.05) was measured. In response to the statin-treatment, a strong reduction (p ≤ 0.05) in iron-oxide-particle accumulation. (**D**) In the scatter-plot, the *in vivo* measured signal-void resulting from the iron-oxide-particles showed a strong correlation (p ≤ 0.05) to the *ex vivo* Perls’-Prussian-blue-stain.

In all mice, including those on high-fat-diet and the statin-treatment, a low CNR was measured in the brachiocephalic vessel-wall at all time-points on non-contrast-enhanced (pre-contrast) MR scans (Fig. 2A). A minor increase in CNR was measured in the two-months’ scans and a strong increase in the four-months’ scans After administration of the elastin-specific MR-probe (Fig. 2A). The measured CNR (Fig. 2A) was lower for mice with the statin treatment and differed to the non-treated four-months-HFD mice. A strong correlation was measured between the CNR and the density of elastic-fibers on Elastica-von-Gieson-staining in the atherosclerotic-plaque (y = 289.2x + 5.8, R² = 0.83, p ≤ 0.05, Fig. 2B).

### T2*-weighted MR imaging for the assessment of macrophage-specific iron-oxide-particles

ApoE^−/−^ and control mice cross-sectional T2*-weighted images of brachiocephalic-artery demonstrated a circulary lumen prior to the administration of the iron-oxide contrast agent ferumoxytol (Fig. 1). Increasing regions of signal-loss were detected in brachiocephalic-plaques in ApoE^−/−^ mice on two and four-months of HFD after 24-hours after ferumoxytol injection. Area of signal-loss was continuous and constituted a substantial percentage of the luminal area. There was no effect detected in the control group. After two and four-months of HFD an increase in percent signal-loss (relative to pre-contrast MRI) was detected with plaque-progression (26.7 ± 8.3%, 47 ± 9.6%, p ≤ 0.05). Less pronounced signal-loss was detected in the statin treatment group compared to animals on four-months of HFD (18 ± 3.6%, p ≤ 0.05). A strong correlation existed between the area loss on T2* and the accumulation of the iron-oxide-particles on Perl’s staining (y = 834.1x + 0.7, R² = 0.88, p ≤ 0.05, Fig. 2D).

### Influence of the gadolinium-based elastin-specific probe on the visualization of iron-oxide-particles and vice versa

**Figure 3:**
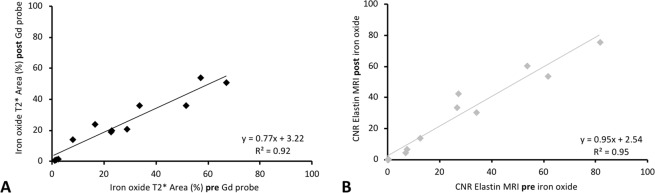
Influence of the gadolinium-based elastin-specific probe on the visualization of iron-oxide-particles and vice versa. If a multi-target approach is chosen, it is of high clinical importance, that the respective imaging probes do not affect the measurements on the other probe. (**A**) To investigate the influence of iron-oxide-particles on the assessment of the elastin-specific-agent on T1-weighted images, imaging was performed on day one prior to the administration of the iron-oxide-particles and 24-hours following the administration of the iron-oxide-particles. CNR-measurements of the elastin-specific MR-probe showed a strong correlation between both days. (**B**) To investigate the influence of the gadolinium-based probe on the assessment of the iron-oxide-particles on T2*-weighted-sequences, imaging was performed on day two prior to and following the administration of the elastin-specific probe. The assessment of the effect of iron-oxide-particles on T2*-weighted images prior to the administration of the gadolinium-based probe demonstrated a strong correlation with measurements following the administration of the gadolinium-based probe.

When using a dual-probe approach, it is of high clinical importance that the gadolinium-based and iron-oxide-based probe do not affect the visualization and quantification of the respective other probe. Therefore, we evaluated the influence of the gadolinium-based probe on the assessment of iron-oxide-particle measurements. 24-hours after the administration of the iron-oxide-particle T2*-weighted-imaging was performed prior to and 45 min following a dose of the elastin-specific probe in animals after four-months of high-fat-diet (n = 9). The assessment of iron-oxide-particles on T2*-weighted images prior to a dose of the gadolinium-based probe demonstrated a strong correlation with measurements following a dose of the gadolinium-based probe (y = 0.77x + 3.2, R² = 0.92, p ≤ 0.05, Fig. 3A). Between both acquisitions no significant differences (p > 0.05) were measured. To investigate the influence of the iron-oxide-particles on the assessment of the elastin-specific agent on T1-weighted images, imaging was performed on day one prior to and 24-hours following the administration of the iron-oxide-particles (n = 9). The CNR measurements of elastin-specific MR-probe showed a strong correlation between both days (y = 0.95x + 2.54, R² = 0.94, Fig. 3B). Between both days no significant difference (p > 0.05) was measured. This indicates that the gadolinium-based and iron-oxide-based probe do not affect the visualization and quantification of the respective other probe at these time-points.

### Histology and Immunofluorescence

#### Elastin in the extracellular-matrix

*Ex-vivo* measurements on histological slices were in good agreement with *in-vivo* measurements using the elastin-specific probe (y = 289.2x + 5.8, R² = 0.83, p ≤ 0.05). After two-months of high-fat-diet, a slight, but already significant (p ≤ 0.05) gain in signal was measured (Fig. 2). After four-months of high-fat-diet, the increase in elastic-fibers in the extracellular matrix of the atherosclerotic-plaque was even stronger (p ≤ 0.05) (Fig. 2) but in response to the statin-treatment, a significant (p ≤ 0.05) decrease in the expression of elastic-fibers was observed (Fig. 2).

#### Macrophages in the atherosclerotic-plaque

**Figure 4:**
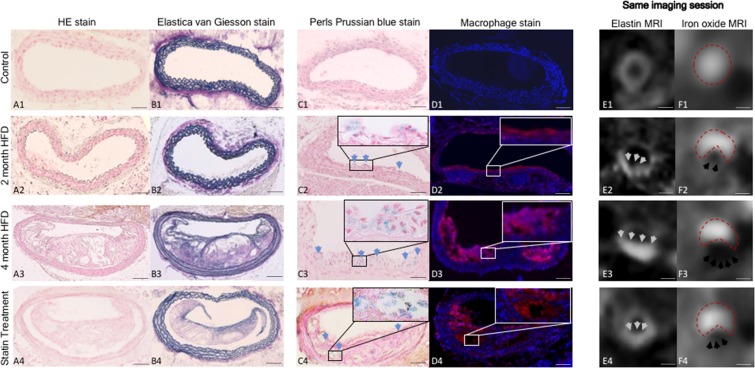
Multitarget characterization of the plaque-burden and inflammatory activity by molecular MRI at different phases of plaque development and in response-to-therapy. (**A–C**) Cross-sectional histological/immunofluorescence sections (A1-4: Elastica-van-Giesson stain, B1-4: Perls’-Prussian-blue-stain, C1-4: Immunofluorescence-macrophage-stain) of brachiocephalic-arteries of control and ApoE^−/−^ mice two-months, four-months of HFD and statin-treatment. (**D,E**) T1 and T2*-weighted-imaging with corresponding histology. D1-4: On T1-weighted sequences, a significant increase of vessel-wall-enhancement is in the vessel wall, corresponding to an increased elastin deposition in atherosclerotic-plaque after four-months of HFD. Only a slight increase can be visualized after two-months and following statin-treatment. E1-4: A pronounced signal-void can already be seen after two-months of high-fat-diet, reflecting an already pronounced accumulation of iron-oxide-particles in the atherosclerotic-plaque. The most pronounced signal-void can be observed after four-months of high-fat-diet with almost 50% of the vascular lumen affected by the signal-void. Following statin-treatment, a comparable signal-void to the two-months group is visualized. (**A–D**) Scale corresponds to 100 µm. (**E,F**) Scale corresponds to 500 µm.

To demonstrated the presence of macrophages in the atherosclerotic-lesions a CD68 staining was used (Fig. 4D1–4). With increasing duration of the HFD an increase in %macrophages and %Perls’ Prussian-blue-staining (R2 = 0.88, p ≤ 0.05) was observed (Fig. 2D). In the statin-group, a significant (p ≤ 0.05) decrease in positive macrophages areas was measured, which was in agreement with a intense decrease in areas positive for iron. Perls’ Prussian-blue-stain was negative for iron-oxide depositions without a prior iron-oxide injection in mice four-months on HFD.

### Gadolinium concentration by inductively-coupled mass-spectrometry (ICP-MS)

**Figure 5:**
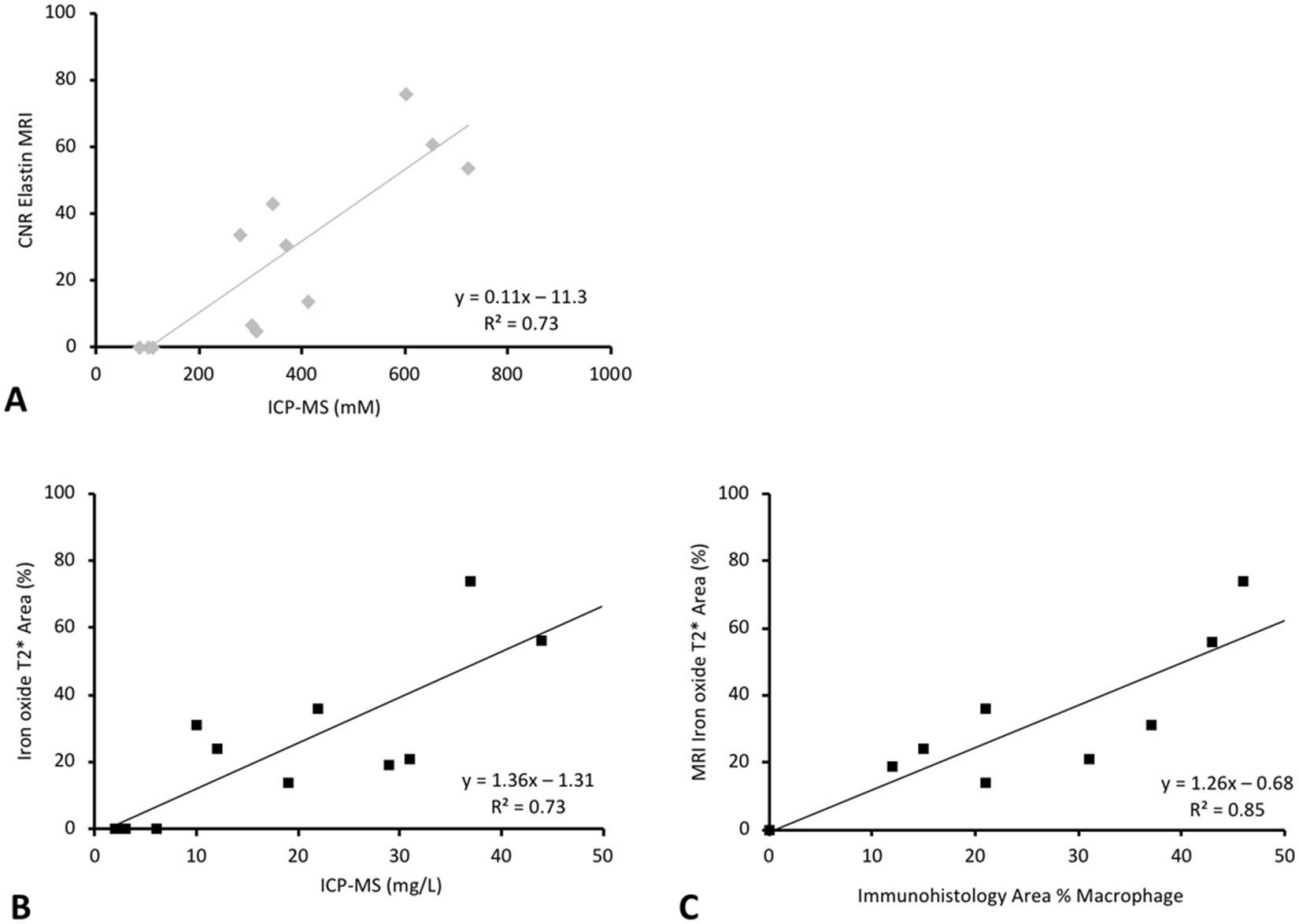
*In vivo* MRI signal measurements and *ex vivo* quantification of molecular probes. (**A**) The scatter-plots show a strong correlation between *in vivo* CNR and results from ICP–MS of brachiocephalic-arteries. (**B**) The scatter-plot shows a strong correlation between the signal-void on the T2*-weighted-sequences with the results from ICP-MS. (**C**) The immunofluorescence-staining of the macrophage area using an antibody (CD68-Antibody) cause a strong correlation (p ≤ 0.05) with the signal-void on the T2*-weighted-sequences.

From early to advanced phases of disease progression the average concentration of gadolinium in the brachiocephalic-artery wall gained substantially (n = 15, Fig. 5). A significant correlation between *ex-vivo* measured gadolinium concentrations (ICP-MS) and CNR (p < 0.05) was found (Fig. 5A). Also, for iron-oxide-particles, a significant correlation between the absolute iron-oxide quantity in the brachiocephalic-artery by ICP-MS and T2* measurements (R2 = 0.73, p < 0.05, Fig. 5B) was observed.

### Spatial localization of the gadolinium-based elastin-specific probe using laser coupled mass spectrometry (LA-ICP-MS)

**Figure 6:**
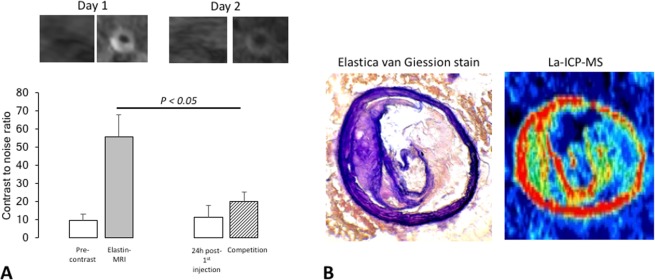
Competition experiments and laser ICP-MS for the mapping of the gadolinium distribution in an atherosclerotic-plaque. (**A**) First a pre-contrast scan was acquired on day one. In the next step 0.2 mmol/kg of the gadolinium-based elastin-specific probe was administered to assess the uptake of the probe in the vessel-wall. After 24-hours, a pre-contrast scan was repeated to confirm the washout of the agent. For the competition experiments, a tenfold-higher dose of non-paramagnetic europium-labeled elastin-specific MR-probe was administered prior to the application of 0.2 mmol/kg of the gadolinium-based elastin-specific probe. This resulted in a marked decrease of the contrast-to-noise-ratio compared to the administration of the gadolinium labeled elastin-specific agent alone. The *in vivo* competition experiments were performed in ApoE^−/−^ mice (n = 3) on a four-months high-fat-diet. (**B**) Spatial localization of the gadolinium-based elastin-specific probe using LA-ICP-MS. Signal from gadolinium across the arterial-wall sample (n = 3) was mapped to determine the distribution of gadolinium. Co–localization of targeted gadolinium with elastic-fibers was found.

We investigated arterial-wall samples of animals after four-months of high-fat-diet (n = 3) and mapped the dispersion of the gadolinium by LA-ICP-MS to define the gadolinium dispersion within the arterial-wall after the administration of the elastin-specific probe,. Co–localization of targeted gadolinium with elastic-fibers was determined (Fig. 6B, n = 3**)**.

### Competition experiments

To reaffirm the specific binding of the elastin-specific molecular agent an *in-vivo* competition experiment was performed. A pre-injection of a tenfold-higher-dose of non-paramagnetic-europium-labeled elastin-specific MR-probe resulted in a distinct decrease of CNR differs to the application of the gadolinium-labeled elastin-specific agent. Between pre-contrast scans on days one and two no significant differences were measured. The *in-vivo* competition experiments were performed in ApoE^−/−^ mice (n = 3) on a four-month’s high-fat-diet (Fig. 6A).

## Discussion

This study demonstrates the feasibility of multi-probe molecular MRI for the simultaneous characterization of extracellular-matrix remodeling and inflammatory activity in a mouse-model of progressive atherosclerosis. There was a temporal delay between de-novo synthesis of elastic-fibers and inflammatory activity in atherosclerotic-plaques. While inflammation was more pronounced in early atherosclerotic-plaques and showed a stronger response to statin therapy, elastin deposition was highest in advanced atherosclerotic-plaques.

This is the first *in-vivo* MR study to demonstrate that both ECM-remodeling and inflammation can be visualized and quantified using a multi-probe approach in a single MR examination. Additionally, it was demonstrated that the gadolinium-based and iron-oxide-based probe do not affect each other and thus visualization and quantification of the respective targets was not compromised.

The simultaneous *in-vivo* detection and quantification of inflammation and ECM-remodeling could be useful for an improved characterization and staging of coronary and carotid atherosclerotic-lesions, which may aid *in-vivo* characterization of the disease, including the assessment of response-to-therapy.

### Multi-probe plaque characterization using an elastin-specific gadolinium-based probe and a macrophage-specific iron-oxide-based probe

Elastic-fibers play an important role during the pathogenesis of atherosclerosis^15^. Besides their elastic properties, elastic-fibers also have molecular signaling functions in atherosclerosis, influencing cell proliferation/migration and the retention of lipoproteins^15^.

Inflammation is considered to be a main drivers of atherosclerosis and especially macrophages are thought to play a central role^16^. Lipoproteins lead to a recruitment of monocytes/macrophages into the subendothelial space, where these cells phagocytose accumulating lipoproteins^17^.

The initiation and progression of atherosclerotic-plaques is a highly complex process, in which various cellular and matrix associated processes occur simultaneously. Different features that are associated with plaque vulnerability can be imaged based on the different available imaging techniques. In recent years, especially the visualization and quantification of vascular-remodeling and inflammation have been shown to represent promising biomarkers for improving the prediction of plaque vulnerability and the associated clinical events^18^.

Atherosclerosis associated vascular-remodeling is a process during which arteries enlarge to compensate for the development of the atherosclerotic-plaque. In the context of atherosclerosis, this process was first described and quantified by Glagov *et al*.^5^. The noninvasive quantification of vascular-remodeling was shown to be a highly promising biomarker to identify early subclinical disease, for the detection of unstable plaque and to monitor the progression and regression of atherosclerosis, e.g. in response-to-therapy. In this context, optical-coherence-tomography (OCT) and intravascular-ultrasound (IVUS) studies demonstrated that positive vascular-remodeling is a relevant feature of vulnerable atherosclerotic-plaques^19^. Previous studies have shown that the elastin-specific gadolinium-based probe used in the present study could be used to visualize elastin in the arterial-wall and that it may be used as a surrogate marker for plaque-burden and vascular-remodeling^11,12^. Gadolinium-based probes are visualized as a result of the shortening of the T1-relaxation-times, resulting in a bright signal on T1-weighted images.

Various preclinical and clinical studies have investigated the use of iron-oxide-particles for the visualization of intraplaque macrophages^20^. In the clinical setting, iron-oxide-particle accumulation in atherosclerotic-plaques correlated with the number of intraplaque macrophages^21^. It was also shown that ruptured or rupture prone plaques show a significantly higher accumulation of iron-oxide-particles compared to stable lesions. Regarding the assessment of response-to-therapy, the ATHEROMA study demonstrated, that the response to statin’s anti-inflammatory effects could be assessed using iron-oxide-particles^22^. Iron-oxide-particles are visualized as a result of the shortening of the local T2/T2*-relaxation-times, creating a detectable signal-void on T2/T2*-weighted images.

For the visualization of the different probes, we used specific sequences. It however has to be considered that T1 and T2 probes also have minor effects on the T2 and T1 relaxation, especially if probes which exhibit a strong effect on both, T1 and T2 relaxation, are used.

This was also the first study, directly investigating the temporal association between intraplaque inflammation and extracellular-matrix remodeling in a single scan. In the current study, we could demonstrate that plaque inflammation and extracellular-matrix remodeling show a different temporal evolution during plaque development. In the early-phase, a pronounced accumulation of iron-oxide-particles was observed, which was in good agreement with a strong inflammatory process as shown by histology. In advanced plaques, a strong expression of elastic-fibers was observed. In response-to-therapy, a pronounced reduction in the accumulation of iron-oxide-particles was measured. These findings indicate that the development of atherosclerotic-plaque in this model is characterized by an early inflammation process, followed by cell migration and finally by extracellular-matrix remodeling (as typically seen in tissue injury such as myocardial infarction). There was no direct temporal association between extracellular-matrix remodeling and inflammation.

The use a two probes at the same time could be associated with challenges regarding a broad clinical translation. However, in the context of PET/MR imaging two probes (e.g. Gadovist and FDG) are also used in combination for tumor detection. The advantage of dual probe MR imaging would be the lack of ionizing radiation in this context.

An additional important finding of this study was that the gadolinium-based and iron-oxide-based probe used in these experiments do not affect the visualization and quantification of the respective other probe, which is especially relevant in a clinical setting.

### Translational potential of this study

The gadolinium-based molecular probe used in this study was administered at a clinical dose and the molecular-weight and signal properties of the probe are comparable to currently used clinical contrast-agents. The applied iron-oxide-particle (also used at a clinical dose) is clinically approved is currently successfully used as an imaging-agent in a clinical setting. Results from this study can be directly translated to human applications, using the same dose and with identical relaxation/rotational-correlation properties of the probes used.

### Mouse-model used in this study

Previous studies have demonstrated that the brachiocephalic-artery of the ApoE^−/−^ mouse-model exhibits a consistent rate of lesion development/progression and that lesions show comparable features as observed during the different stages of plaque development in humans^23^. A main limitation of animal models in general is, that they never fully resemble human disease.

## Conclusion

This study demonstrates the feasibility of a simultaneous dual-probe molecular MRI for the simultaneous characterization of plaque-burden and inflammatory activity by in a mouse-model of progressive atherosclerosis. The *in-vivo* detection and quantification of these biomarkers associated with plaque instability in a single scan may enable the detection of potentially unstable plaque and thereby improve risk stratification and guidance of treatment and allow to monitor treatment response.

## Supplementary information

revised supplementary material

## Data Availability

The datasets generated and analysed during the current study are available from the corresponding author on reasonable request.
