# Supplementary material for: Dual-probe molecular MRI for the in vivo characterization of atherosclerosis in a mouse model: Simultaneous assessment of plaque inflammation and extracellular-matrix remodeling
Source: Sci Rep. 2019 Sep 25;9:13827. doi: 10.1038/s41598-019-50100-8 (PMC6761132; doi:10.1038/s41598-019-50100-8)
Supplement: Supplementary file 1 — revised supplementary material [file 41598_2019_50100_MOESM1_ESM.docx]

Dual-probe molecular MRI for the in vivo characterization of atherosclerosis in a mouse model:  *Simultaneous assessment of plaque inflammation and extracellular-matrix remodeling*

*Carolin Reimann, VMD^a,b^, Julia Brangsch, VMD^a,b^, Jan O. Kaufmann^a,c,d^,

Lisa C. Adams, MD^a^, David C. Onthank, PhD^e^, Christa Thöne-Reineke, PhD^b^,

Simon P. Robinson, PhD^e^, Bernd Hamm, MD^a^,

Rene M. Botnar, PhD^f,g,h,i^, Marcus R. Makowski, MD^a,f,h^

**Supplementary material**

**Materials and Methods**

**Animals**

Mice were housed under controlled conditions (12:12 h light/dark cycle, lights on 07:00 h, temperature, 22 ± 2 °C) and provided with food and water ad libitum. Eight weeks old homozygous apolipoprotein E-deficient (ApoE^-/-^) male mice were used (Forschungseinrichtung für experimentelle Medizin (FEM), Berlin, Germany). For imaging controls, nine 26 week old male homozygous C57BL/6J mice from Charles River Laboratories (Sulzfeld, Germany) were used.

All animals were housed in a clean barrier. Prior to imaging sessions, mice were anesthetized using an intraperitoneal administration of a combination of Medetomidin (500 µg/kg), Fentanyl (50 µg/kg) and Midazolam (5 mg/kg). For serial imaging experiments, a reversal agent, consisting of Atipamezol (2.5 mg/kg), Flumazenil (500 µg/kg) and Naloxon (1200 µg/kg), was administered after the imaging session.(1) Following the final imaging session, mice were euthanized. For histological examinations, a perfusion with the fixative MorFFFix® (Morphisto, Frankfurt am Main, Germany) at a pressure of 100 mm Hg was performed followed by excision of the carotid arteries, brachiocephalic artery and aortic artery. All animal procedures were carried out by a veterinarian and all possible steps were taken to avoid animal suffering at all time points of the experiments.

***In Vivo* MR Experiments**

For the administration of the different MR imaging-agents, a small diameter tube with an attached needle was inserted into the tail vein of the animals. During the imaging sessions, body temperature (37 °C) was monitored using a MR-compatible heating system (Model 1025, SA Instruments Inc, Stony Brook, NY).

*Elastin imaging using T1-weighted sequences*

For localization of the aortic artery, brachiocephalic artery and carotid arteries, a low-resolution three-dimensional (3D) localizer scan was performed in sagittal, coronal and transverse orientation using the following parameters: field-of-view (FOV) = 280 mm, matrix = 320, slice thickness = 3 mm, TR/TE = 7.7/3.7 ms, flip angle = 20° and slices = 10. The scout scan was followed by a two-dimensional (2D) time-of-flight (TOF) scan in transverse orientation for visualization of the aortic arch and the brachiocephalic artery. Imaging parameters included: FOV = 200 mm, matrix = 960, in plane spatial resolution = 0.2 x 0.2 mm, slice thickness = 500µm, TR/TE = 35/4.5 ms, flip angle = 90° and slices = 26. From the TOF dataset, a maximum intensity projection (MIP) was generated for display of an arterial angiogram of the aortic arch, the brachiocephalic artery and the carotid arteries to plan the subsequent contrast-enhanced sequences. The inversion recovery scan to visualize the gadolinium-based contrast agent was preceded by a 2D Look-Locker sequence planned perpendicular to the ascending aorta, which was used to determine the optimal inversion time (TI) for blood signal nulling. Imaging parameters of the Lock Locker sequence included: FOV = 300 mm, matrix = 750, in plane spatial resolution = 0.4 x 0.4 mm, slice thickness = 1.5 mm, TR between subsequent IR pulses = 1000 ms, and flip angle = 15°. Imaging parameters of the high-resolution 3D inversion recovery gradient echo late gadolinium enhancement (LGE) sequence scan employed for visualization of gadolinium-based molecular probe were: FOV = 57 mm, matrix = 416, inplane spatial resolution = 0.137 x 0.137 mm, slice thickness = 370 µm, slices = 56, TR/TE = 12.1/5.7 ms, TR between subsequent IR pulses = 1000 ms, and flip angle = 30°.

*Iron oxide imaging using T2*-weighted sequences*

Imaging parameters for the T2* weighted sequences in this study included a field of view 150 x 150 mm; matrix 832 x 832; in-plane spatial resolution 0.18 x 0.18 mm; slice thickness 500 µm; TR/TE 17/7.4 ms; flip angle 20°; averages, Phase Partial Fourier 6/8, and 32 slices.

**Histological analysis and immunofluorescence of the arterial vessel system and plaque morphometry**

Histological analysis of the brachiocephalic artery was performed as described below. The vessels were embedded in paraffin and cut into 5 µm thick serial sections. These sections were dewaxed and stained with Miller’s Elastica-van-Gieson-stain (EvG) for visualization of elastin and Perls‘ Prussian-blue-stain for the visualization of iron-oxide-particles. Additionally, a standard Hematoxylin-and-Eosin (HE) stain was performed. For co-registration of histological sections and MR images, the aortic arch and the subclavian artery were use as landmarks. Analyses were made on digitized images of EvG, Perls‘ Prussian blue and immunofluorescence sections. Morphometry as well as quantification of staining area were measured using computer-assisted image-analysis (ImageJ software, Version 1.51).

Immunofluorescence staining of macrophages using a primary antibody (rat anti mouse CD68, Bio-Rad, 1:100) and Dako REAL^TM^ Antibody Diluent (Dako, Denmark) required incubation overnight at 8 degrees. Sections were washed two times with PBS, pH 7.4. Macrophages binding was located by incubation with the polyclonal secondary Antibody (goat anti-rat IgG, Thermo Fisher Scientific, Germany, 1:200). Slides were washed three times with PBS, pH 7.4, counterstained and mounted with Roti^®^-Mount FlourCare (Carl Roth, Germany). Co-registration of macrophages on CD68 staining and Perls‘ Prussian blue positive staining areas was assessed in serial sections of atherosclerotic lesions.

**Inductively coupled mass spectrometry for quantification of gadolinium and iron and Laser-inductively-coupled-mass-spectrometry (LA ICP-MS) for spatial localization of gadolinium**

Inductively coupled mass spectrometry (ICP–MS) was conducted using vessel tissue samples at each time point (n=3 per group). After the last imaging session, brachiocephalic samples were digested at 37°C in 70% nitric acid overnight, followed by dilution with deionized water to an acid concentration of 2.5% for ICP–MS analysis. For each sample set a standard curve was documented for iron oxide and gadolinium concentration.

Brachiocephalic arteries were cut at -20 °C into 10 µm cryosections and immediately mounted on SuperFrost Plus adhesion slides (Thermo Scientific). The laser ablation was performed with a laser ablation system NWR 213 (New Wave Research, Fremont, CA, USA) connected to an inductively coupled plasma mass spectrometer (Agilent 7900, Agilent Technologies, Japan) using line scans with a laser energy of 34%, a spot size of 20 µm and a measurement speed of 20 µm/s. For quantification of the Gadolinium (Gd) amount in brachiocephalic arteries, rat brain standards with well-defined Gd concentration were analyzed under the same conditions. The calibration, the reconstruction, and the visualization of the images were performed by an in-house developed software (Forschungszentrum Jülich, Jülich, Germany).Methods were established as mentioned in our previous studies (Supplementary References 1-3).

Competition experiments

*In vivo* competition experiments were performed in ApoE^-/-^ mice (n = 3) on a four months high-fat-diet. The elastin-specific probe, ESMA, was administered on day one at a clinically relevant dose of 0.2 mmol/kg. After the day one scanning session, a preinjection of a tenfold higher dose of the non-paramagnetic europium-labeled elastin-specific MR probe was performed. On day two, the elastin-specific probe was administered at a clinically relevant dose of 0.2 mmol/kg. All contrast agents were applied *via* the tail vein.

**Statistical Analysis**

Values are specified as mean ± standard deviation. Values of the treatment and control group were compared using SigmaStat 4.0 (Systat Software, Inc). For the comparison of continuous variables, a Student’s t test (unpaired, two–tailed) was applied. In case of more than two groups, statistical comparisons were performed by analysis of variance (ANOVA) followed by the Bonferroni test. p<0.05 was regarded to be statistically significant.

**Supplementary References:**

1. Makowski MR, Wiethoff AJ, Blume U, Cuello F, Warley A, Jansen CH, et al. Assessment of atherosclerotic plaque burden with an elastin-specific magnetic resonance contrast agent. Nat Med. 2011;17(3):383-8.

2. Makowski MR, Preissel A, von Bary C, Warley A, Schachoff S, Keithan A, et al. Three-dimensional imaging of the aortic vessel wall using an elastin-specific magnetic resonance contrast agent. Invest Radiol. 2012;47(7):438-44.

3. Reimann C, Brangsch J, Kaufmann JO, Adams LC, Onthank DC, Robinson SP, et al. Contrast-Enhanced Magnetic Resonance Angiography Using a Novel Elastin-Specific Molecular Probe in an Experimental Animal Model. Contrast Media Mol Imaging. 2018;2018:9217456

**Supplementary Figure Legends**

**Supplementary Figure 1:** **Experimental setup of the study.**

The animal was imaged at two time points, on day one and after 24 hours on day two. On day one, first a pre-contrast MRI with a T1 and T2*-weighted sequence was performed. Then the elastin-specific probe was injected at the clinical dose of 0.2 mmol/kg. After 40 minutes the T1-weighted MR sequences were performed. After the imaging session was finished, the iron oxide particle (ferumoxytol) was injected at a clinical dose of 4mg Fe/kg. On day two, first a native T1-weighted MR sequence was performed to exclude any residual binding of the elastin agent in the vessel wall. Then the T2*-weighted sequence was acquired to measure the accumulation of the iron oxide particles in the vascular wall. Following that the elastin-specific probe was injected for a second time. After 40 minutes, a T1-weighted MR sequence was performed to assess the uptake of the probe in the vessel wall. Following this, an additional T2*-weighted sequence was performed to exclude any effect of the gadolinium-based probe on the T2* images. Following the final imaging session, the brachiocephalic artery was surgically removed for further *ex vivo* analysis.

**Supplementary Figures**

Supplementary Figure 1

**
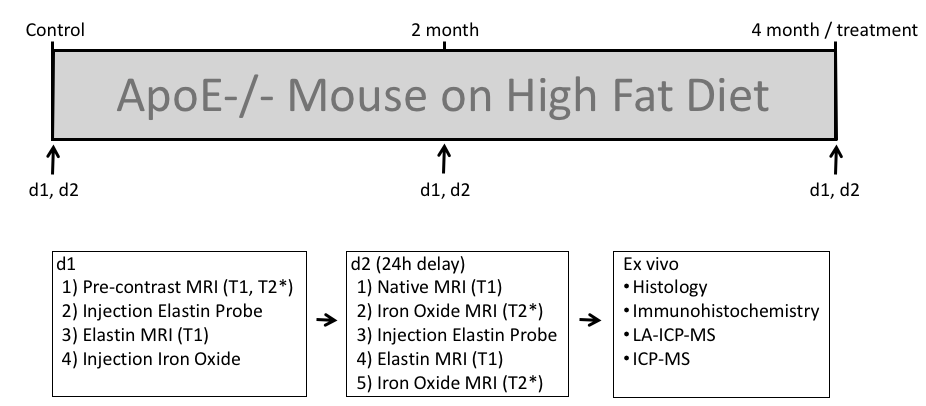
**
